# Spontaneous Tension Pneumothorax as a Complication of COVID-19

**DOI:** 10.1155/2021/4126861

**Published:** 2021-01-04

**Authors:** Muhammad Umar Shahzad, Jennie Han, Mohammad Ishack Ramtoola, Vasileious Lamprou, Urmi Gupta

**Affiliations:** ^1^Department of Cardiology, Royal Lancaster Infirmary, Lancaster LA1 4RP, UK; ^2^Department of Respiratory Medicine, Royal Lancaster Infirmary, Lancaster LA1 4RP, UK

## Abstract

*Key Clinical Message*. Tension pneumothorax is an uncommon presentation in patients with SARS-CoV-2 (severe acute respiratory syndrome coronavirus 2) or COVID-19 pneumonia. We present a case of tension pneumothorax in a patient with COVID-19 pneumonia and myocarditis. This was an unlikely diagnosis in a patient with no known underlying lung condition and no other precipitating factors such as barotrauma. In an acute deterioration of patients with SARS-CoV-2, it is important to always consider alternative diagnoses and repeat imaging.

## 1. Introduction

Severe acute respiratory syndrome coronavirus 2 (SARS-CoV-2) is a novel coronavirus which causes coronavirus disease 2019 (COVID-19). It was first identified amid an outbreak of respiratory illness cases in Wuhan City, Hubei Province, China [1]. COVID-19 was reported to the World Health Organisation (WHO) on 31^st^ December 2019 and declared as a global pandemic on 11^th^ March 2020 [1]. Presentations of COVID-19 range from asymptomatic or mild symptoms to severe illness or death. Multiple complications of COVID-19 including acute respiratory distress syndrome (ARDS) [2, 3], acute respiratory failure (ARF) [2, 3], myocarditis, heart failure, arrhythmias, acute coronary syndrome (ACS) [4, 5], disseminated intravascular coagulation (DIC) [6–8], venous thromboembolism [6–8], secondary infection [2, 3], acute kidney injury [2, 3], pancreatic injury [8], and neurological complications such as seizures, meningitis, encephalitis, encephalopathy, ataxia, and neuralgia [9] have been reported in the literature thus far.

Pneumothorax is a common respiratory condition which is defined as the presence of air in the pleural cavity. Pneumothorax can be categorised by aetiology into spontaneous and traumatic, and spontaneous pneumothorax can be further characterised into primary or secondary. Primary spontaneous pneumothorax occurs in patients with no background of respiratory disease, and the pathophysiology is thought to be from asymptomatic blebs and bullae which disrupt due to shear force [10]. Secondary pneumothorax occurs in patients with underlying lung abnormality and diseases including asthma, chronic obstructive pulmonary disease, cystic fibrosis, lung cancer, pulmonary fibrosis, extrinsic allergic alveolitis, sarcoidosis, pneumonia, tuberculosis, acute respiratory distress syndrome, and connective tissue disorders. Many cases of traumatic pneumothorax are iatrogenic, and this complication has been reported in central vein cannulation, pleural tap or biopsy, transbronchial biopsy, fine needle aspiration, and acupuncture [11]. Pulmonary barotrauma is another cause of iatrogenic pneumothorax occurring in patients being mechanically ventilated due to high inspiratory inflation pressures.

Tension pneumothorax develops when damaged tissue forms a one-way valve leading into the accumulation of air in the pleural space with inhalation. This results in rising volume of air and pressure in the affected hemithorax, leading to collapse of the affected lung and displacement of the mediastinum towards the contralateral side. This is a medical emergency, as the mediastinal shift exerts pressure on the contralateral lung and the vena cava, resulting in respiratory insufficiency, cardiovascular compromise, and death if untreated.

## 2. Case Report

We present the case of a 62-year-old gentleman, who was brought into the emergency department (ED) by ambulance with a history of dry cough, low grade fever, worsening shortness of breath for 4 days, and pleuritic sounding chest pain, which was different to when he had a pulmonary embolism approximately ten months ago. His past medical history included type II diabetes mellitus, peripheral vascular disease, treated malignant neoplasm of the base of tongue, diverticular disease, essential hypertension, and previous provoked pulmonary embolism. Before admission, he was living independently with his wife. His exercise tolerance was restricted to 10 yards with the assistance of a walking stick. He was an exsmoker and used to smoke 5 cigarettes a day but stopped smoking a few years previously.

**Figure 1:**
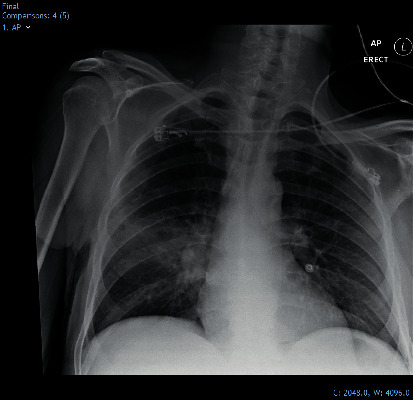
Chest radiograph on admission demonstrating right midzone consolidation.

On admission, he was pyrexial with a temperature of 38.2°C, oxygen saturations 98% on 28% FiO_2_, blood pressure 73/35 mmHg, and pulse rate 92 bpm. Initial treatment included oxygen 28% FiO_2_ via a venturi mask and fluid resuscitation for hypotension. Physical examination revealed bibasal crepitations on auscultation of chest and a swollen and tender right leg. Initial investigations revealed deranged biochemical markers (Table 1) and opacification affecting the right midzone of the lung (Figure 1).

Initial laboratory workup summarised in Table 1.

**Figure 2:**
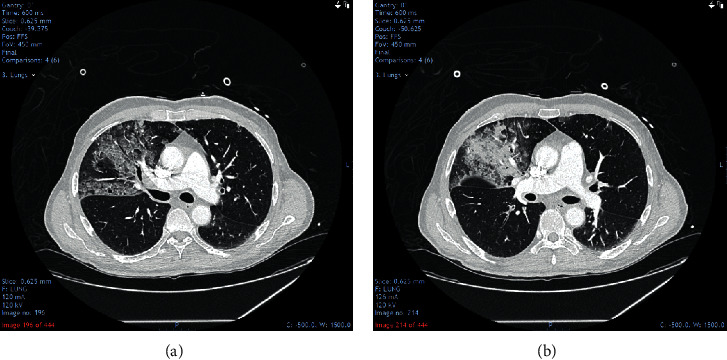
CT pulmonary angiogram report: there are no pulmonary emboli and pleural or pericardial effusions. There is consolidation within the right upper lobe with subtle peripheral ground glass opacification noted in the left upper lobe and in both lower lobes. Appearances are indeterminate but could represent early COVID-19 disease. The central and peripheral airways are patent with no lower cervical, axillary, and mediastinal lymphadenopathy and lung mass. Other viscera are unremarkable.

In view of the clinical presentation and raised d-dimer, a computed tomography pulmonary angiogram (CTPA) was performed which reported right upper lobe consolidation with subtle peripheral ground glass opacification in the left upper lobe and both lower lobes, suggestive of early COVID-19 infection (Figure 2). Moreover, his troponin I levels were markedly elevated and electrocardiogram (ECG) showed ST segment depression and T-wave inversion in the anterolateral chest leads. A transthoracic echocardiogram showed moderately impaired left ventricular systolic function with an akinetic and rounded apical septal wall.

On account of the history, examination, and investigations, he was treated as suspected COVID-19, community-acquired pneumonia (CURB-65 score = 2, moderate severity), COVID-19 myocarditis, and sepsis, leading to multiorgan failure. In view of poor physiological reserve and comorbidities, the ceiling of care was decided to be ward level care and noninvasive ventilation if needed. However, he began to improve clinically, became normotensive, and was able to be weaned off oxygen day 3 after admission. By that time, his swab detected SARS-CoV-2 RNA which confirmed the diagnosis of COVID-19.

**Figure 3:**
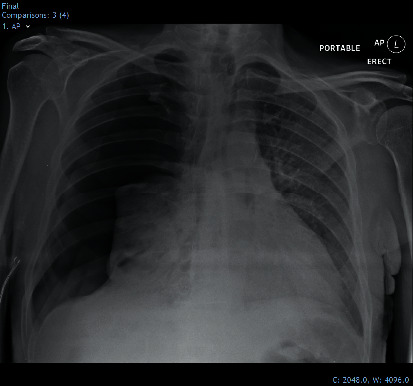
Portable chest radiograph following acute deterioration, demonstrating a right-sided tension pneumothorax.

Following initial improvement, he suddenly developed a new, increased oxygen demand, requiring titration of humidified oxygen up to 10 litres. Chest physiotherapy helped him bring up copious volumes of thick sputum, and he was stable for most of the day with oxygen saturations of 93%. Later that day, he suddenly deteriorated and becoming cyanotic and critically unwell. Oxygen saturations were 80% on 15 litres nonrebreathe mask, and he was significantly tachypnoeic with a respiratory rate of 40/minute. An urgent portable chest X-ray revealed a large right-sided pneumothorax causing mediastinal shift (Figure 3).

An intercostal drain was inserted on an emergency basis and the patient improved slightly over the next 24 hours though he still required oxygen via a high flow nasal cannula system to maintain oxygen saturations of 92% or more. Despite the chest drain and the addition of thoracic suction, the lung did not reexpand. He continued to deteriorate rapidly, was unable to tolerate further pleural procedures, and died.

## 3. Discussion

Here, we present a case of spontaneous tension pneumothorax in the setting of COVID-19 pneumonia and myocarditis. Although our patient did not have a confirmed diagnosis of chronic obstructive pulmonary disease before the admission, review of his CTPA images revealed minimal emphysematous changes in the right lung which could have ruptured by the added insult of COVID-19 pneumonia. This could have led to an air leak into the hemithorax, resulting in a spontaneous tension pneumothorax.

COVID-19 is a newly described disease with limited and ever-expanding knowledge of its clinical manifestations. Multiple causes of acute clinical deterioration have been already described, including ARDS and pulmonary embolism [12]. This case report aims to highlight the importance of urgent chest X-ray and prompt clinical examination in patients with COVID-19 that develop a sudden increase in oxygen requirements and become acutely tachypnoeic in order to rule out the possibility of pneumothorax. In view of the lack of a formal diagnosis of previous lung disease, this situation could have been misinterpreted as progression of ARDS in the current climate. This could have resulted in further deterioration of the patient if CPAP had been introduced [13]. Furthermore, chest physiotherapy is contraindicated in untreated tension pneumothorax, and depending on the time frame of pneumothorax development, may have had a negative effect on the clinical course of this patient.

Two recent case reports have been published regarding the link between COVID-19 and spontaneous pneumothorax; however, the absence of published literature of such cases could result in clinicians missing these diagnoses; hence, we present this case [14, 15].

## 4. Conclusion

In summary, our case demonstrates spontaneous tension pneumothorax as a rare but serious complication of COVID-19. Meticulous physical examination and repeat sequential chest X-rays can be useful tools in the diagnosis of complications and management of disease.

## Figures and Tables

**Table 1:** Blood results.

Blood	Result	Normal reference range
CRP	123 mg/L	<5 mg/L
Pro-calcitonin	56.81 ng/ml	<0.5 ng/ml
Ferritin	624 *µ*g/L	24–250 *µ*g/L
Hb	102 g/L	125–180 g/L
WBC	4.6 × 10^9^/L	4.0–10.0 × 10^9^/L
Neutrophils	4.1 × 10^9^/L	2.0–7.5 × 10^9^/L
Lymphocytes	0.2 × 10^9^/L	1.0–3.0 × 10^9^/L
D-Dimer	879 ng/ml	0–278 ng/ml
Troponin I-1^st^	7241 ng/L	<20 ng/L
Troponin I-2^nd^	10 474 ng/L	<20 ng/L
